# Antinuclear antibodies produced in HLA-DR transgenic humanized mice developed chronic graft-versus-host disease

**DOI:** 10.1016/j.heliyon.2021.e08380

**Published:** 2021-11-11

**Authors:** Hiroshi Tsuzuki, Yasuko Nagatsuka, Mitsuhiro Iwata, Noboru Kitamura, Yosuke Nagasawa, Taro Matsumoto, Ryoji Ito, Takeshi Takahashi, Mamoru Ito, Hideki Nakamura, Masami Takei

**Affiliations:** a Division of Hematology and Rheumatology, Department of Medicine, Nihon University School of Medicine, Tokyo, Japan; b Division of Cell Regeneration and Transplantation, Department of Functional Morphology, Nihon University School of Medicine, Tokyo, Japan; c Central Institute for Experimental Animals, Kawasaki, Japan

**Keywords:** Chronic GVHD, HLA-DR transgenic humanized mouse, Autoantibody, Antinuclear antibody

## Abstract

**Background:**

Chronic graft versus host disease (GVHD) has been reported in humanized mice after the implantation of human hematopoietic stem cells (hu-HSC). As such, humanized mice have been applied to a mouse model of chronic GVHD; however, B-cell activation and autoantibody production did not occur, and the clinical features of chronic GVHD were not sufficiently reproduced. The purpose of this study was to establish an improved humanized mouse model with chronic GVHD using HLA-DR transgenic NOD/Shi-scid, IL-2RγKO (NOG) mice.

**Methods:**

CD34-positive cells were isolated from blood extracted from HLA-DRB1∗0405-positive umbilical cords using magnetic cell isolation. Then these were transplanted into NOG-Iab KO, HLA-DR 0405 Tg mice aged 8–16 weeks. GVHD symptoms were observed 26 weeks after transplantation. Histological findings of the skin, lung, liver, and spleen were compared with those of non-humanized mice. Antinuclear antibodies (ANA) were measured by indirect immunofluorescence using sera isolated 26 weeks after transplantation.

**Results:**

Although GVHD symptoms were not observed in humanized (hu-HSC) NOG-Iab KO, HLA-DR 0405 Tg mice during the observation period, histological findings of human T-cell infiltration were observed in the skin, liver, and lung, suggesting that GVDH was present; human tingible body macrophages or clusters of BCL-6-positive human B-cells were observed in the spleen. Furthermore, human IgG ANA with peripheral or homogeneous staining patterns were also detected in the sera.

**Conclusion:**

Hu-HSC NOG-Iab KO, HLA-DR 0405 Tg mice differed from conventional models in terms of B-cell activation and ANA production. This study is the first to report on B-cell activation and autoantibody production in humanized mice with chronic GVHD, suggesting that hu-HSC NOG-Iab KO, HLA-DR 0405 Tg mice could be applied to a new humanized mouse model of chronic GVHD.

## 1 Introduction

Graft versus host disease (GVHD) is a complication unique to allogeneic hematopoietic stem cell transplantation. Chronic GVHD affects 30–50% of patients who have undergone allogeneic hematopoietic stem cell transplants [1] and accounts for 5–11% of late deaths [2, 3]. This is especially relevant in understanding the long-term survival of patients after transplantation. Chronic GVHD is characterized by symptoms common to autoimmune diseases, such as scleroderma, Sjogren's syndrome, myositis, autoimmune hemolytic anemia, and immune thrombocytopenic purpura [4]. It has been reported that antinuclear antibodies (ANA), such as anti-DNA antibodies and antinuclear body antibodies, were detected in the serum of patients with chronic GVHD [5, 6, 7].

Recently, xenograft humanized mice models have been used in the study of chronic GVHD. Sonntag [8] reported that chronic GVHD-like changes, such as gingivitis and hair loss, occurred in humanized mice produced via the transplanting human hematopoietic stem cells (hu-HSC) into the NOD/SCID/IL-2Rγ^null^ (NSG) mice. Additionally, Ono [9] reported that in humanized mice, produced via the transplantation of hu-HSCs into human IL-6 transgenic NSG mice, the subsequent overproduction of IL-6 activated T-cells and macrophages, resulting in late-onset lethal GVHD. However, in chronic GVHD mice, the clinical features involved in the humoral immune system, such as in B-cell activation or the production of autoantibodies, have not been reproduced.

NSG mice were obtained by mating NOD/SCID mice with IL-2 receptor γ chain deficient mice [10]; these are similar to NOD/Shi-scid, IL-2RγKO (NOG) mice in terms of immunological characteristics [11]. Furthermore, it has been reported that IgG antibodies against foreign antigens are not produced, and reconstruction of the humoral immune system is incomplete in these humanized mice [12]. It is speculated that in Hu-HSC NOG mice, human CD4-positive T-cells have a low affinity for MHC class II of human B-cells because human T-cells with an affinity for MHC class II molecules of mouse thymic epithelium undergo positive selection during the differentiation of human T-cells. Suzuki [13] developed NOG-Iab KO, HLA-DR 0405 Tg mice, and reported that these mice transplanted with CD34-positive cells isolated from HLA-DRB1∗0405-positive umbilical cord blood (UCB) produced IgG antibodies against foreign antigens, although the mice transplanted with CD34-positive cells isolated from HLA-DRB1∗0405-negative UCB did not produce IgG antibodies against foreign antigens. In other words, humanized mice produced by transplanting HLA-DRB1∗0405-positive HSC into NOG-Iab KO, HLA-DR 0405 Tg mice presented better reconstruction of the humoral immune system than conventional NOG mice. Therefore, it is possible that the clinical features of chronic GVHD, especially B-cell activation or autoantibody production, can be reproduced more accurately in hu-HSC NOG-Iab KO, HLA-DR 0405 Tg mice.

Here, we report that in hu-HSC NOG-Iab KO, HLA-DR 0405 Tg mice, chronic GVHD-like changes in the skin, lungs, and liver, B-cell activation, and ANA production occur. The findings of the humoral immune system have not been reproduced previously in humanized mice. Therefore, this study suggests that hu-HSC NOG-Iab KO, HLA-DR 0405 Tg mice can be utilized in studying chronic GVHD.

## 2 Materials and methods

### 2.1 Isolation of CD34-positive hematopoietic stem cells

This study was performed according to the ethical guidelines approved by the Research Ethics Committee at Nihon University School of Medicine (permission number: 211-3). HLA-DRB1∗0405-positive frozen UCB was obtained from the Department of Functional Morphology, Division of Cell Regeneration and Transplantation, Nihon University School of Medicine. Frozen UCB was thawed, and the cell suspension was washed using MACS Buffer (Miltenyi Biotec, Bergisch Gladbach, Germany). A blocking reagent for human Fc receptor (Miltenyi Biotec) and CD34 microbeads (Miltenyi Biotec) were added to the washed cell suspension for subsequent cell labeling. CD34-positive cells were separated using a QuadroMACSTM separator (Miltenyi Biotec). After isolation, CD34-positive cells were suspended in 150 μL of phosphate-buffered saline (PBS) and immediately transplanted into NOG-Iab KO, HLA-DR 0405 Tg mice. The purity and viability of the isolated CD34-positive cells were >95% and >90%, respectively.

### 2.2 Production of hu-HSC NOG-Iab KO, HLA-DR 0405 Tg mice

NOG-Iab KO, HLA-DR 0405 Tg mice (NOD.Cg-Prkdc<scid> Il2rg<tm1Sug> H2-Ab1<tmDoi> Tg(IE-HLA-DRA∗0101/IE-HLA-DRB1∗0405)>/Jic mice) were obtained from the Central Institute for Experimental Animals (Kawasaki, Japan) and bred under specific pathogen-free conditions. CD34-positive cells, suspended in PBS, were administered to 8–16-week-old NOG-Iab KO, HLA-DR 0405 Tg mice via the tail vein. The number of transplanted CD34-positive cells was between 5.8 × 10^4^ and 2.7 × 10^5^ per mouse. In this study, the mice were not irradiated before transplantation. After transplanting CD34-positive cells into the mice, we observed them for signs of GVHD, such as hair loss, weight loss, rounded back, bristling hair, slow movement, tachypnea, gingival inflammation, and tooth weakening, for 26 weeks after transplantation [8, 9, 14]. The mice were euthanized and dissected at the end of the study. All animal experiments were approved by the Animal Experiment Committee of Nihon University (Permission number: AP18MED009-5 and AP18MED010-3) and complied with the ARRIVE guidelines.

### 2.3 Multicolor flow cytometry

Peripheral blood was collected via either tail vein puncture or cardiac puncture. Fetal bovine serum (FBS, 2%; Thermo Fisher Scientific, Waltham, MA, USA) in PBS was combined with heparin-added peripheral blood with the antibody reagent described below. The mixture was allowed to react in a cool and dark place for 30 min. After this reaction, OptiLyse® C (Beckman Coulter, Brea, CA, USA) was added, and then hemolysis and fixation were performed in the dark at room temperature (20°C–25 °C) for 15 min. After hemolysis and fixation, the sample was washed with PBS, and the cell suspension was analyzed using Cytomics FC 500 (Beckman Coulter). The following antibodies were used: anti-human CD3-phycoerythrin-Texas Red-x (ECD, Clone: UCHT1, Beckman Coulter), anti-human CD4-r-phycoerythrin (PE, clone: RPA-T4, Becton Dickinson, San Jose, CA, USA), anti-human CD8-fluorescein isothiocyanate (FITC, clone: RPA-T8, Becton Dickinson), anti-human CD19-phycoerythrin-Cy7 (PC7, Clone: J3-119, Beckman Coulter), anti-human CD34-PE (clone: 581, BD Pharmigen™, San Jose, CA, USA), and anti-human CD45-phycoerythrin-Cy5 (PC5, Clone: J33, Beckman Coulter).

### 2.4 Enzyme-linked immunosorbent assay

Human immunoglobulin levels in mice sera were measured using commercially available kits: Human IgG enzyme-Linked ImmunoSorbent assay (ELISA) kit and Human IgM ELISA kit (Bethyl, Montgomery, TX, USA).

### 2.5 Immunohistochemistry

The mice were euthanized by cervical dislocation; the skin, lungs, liver, spleen, and gastrointestinal tract were harvested. Each organ was fixed with 10% buffered formalin solution and embedded in paraffin. Paraffin sections were deparaffinized and stained with hematoxylin and eosin.

Immunostaining was performed using the following monoclonal primary antibodies: anti-human CD3 (Clone: PS1, BioGenex, San Ramon, CA, USA), anti-human CD19 (Clone, EP169, BioGenex), anti-human CD4 (Clone: 4B12, BioGenex), anti-human CD8 (Clone: SP16, BioGenex), anti-human CD68 (Clone: CD68/G2, BioGenex), and anti-human BCL-6 (Clone: LN22, BIOCARE Medical, Pacheco, CA, USA). Secondary antibody reactions either utilized the Histo-fine Mouse Stain kit (Nichirei) or Histo-fine Simple Stain Mouse MAX-PO (R) (Nichirei). 3,3′-Diaminobenzidine (Nichirei) was used as the chromogenic substrate. Hematoxylin counterstaining was subsequently performed.

### 2.6 Cell culture

HeLa cells (RIKEN RCB007) were purchased from RIKEN BRC (Ibaraki, Japan) and grown in monolayer cultures at 37 °C and with 5% CO_2_ in Dulbecco's Modified Eagle Medium (Sigma-Aldrich, St. Louis, MO, USA), supplemented with 10% FBS, 100 U/mL penicillin (Nacalai Tesque, Kyoto, Japan), and 100 μg/mL streptomycin (Nacalai).

### 2.7 Indirect immunofluorescence microscopy

Mouse sera were tested by indirect immunofluorescence using HeLa cells grown on glass covers. The cells were washed in PBS, fixed with 10% buffered formalin, permeabilized, and blocked. PBS containing 5% FBS (Thermo Fisher Scientific), 2% bovine serum albumin (Thermo Fisher Scientific), and 0.4% Triton X-100 (Sigma-Aldrich) were used for the permeabilization and blocking steps. The cells were first incubated with PBS-diluted sera and PBS-diluted Alexa Fluor® 488 conjugated anti-human IgG (Jackson ImmunoResearch, West Grove, PA, USA). PBS-diluted sera of non-humanized NOG-Iab KO, HLA-DR 0405 Tg mice were used as the negative control.

## 3 Results

### 3.1 Immunohistochemistry

**Figure 1:**
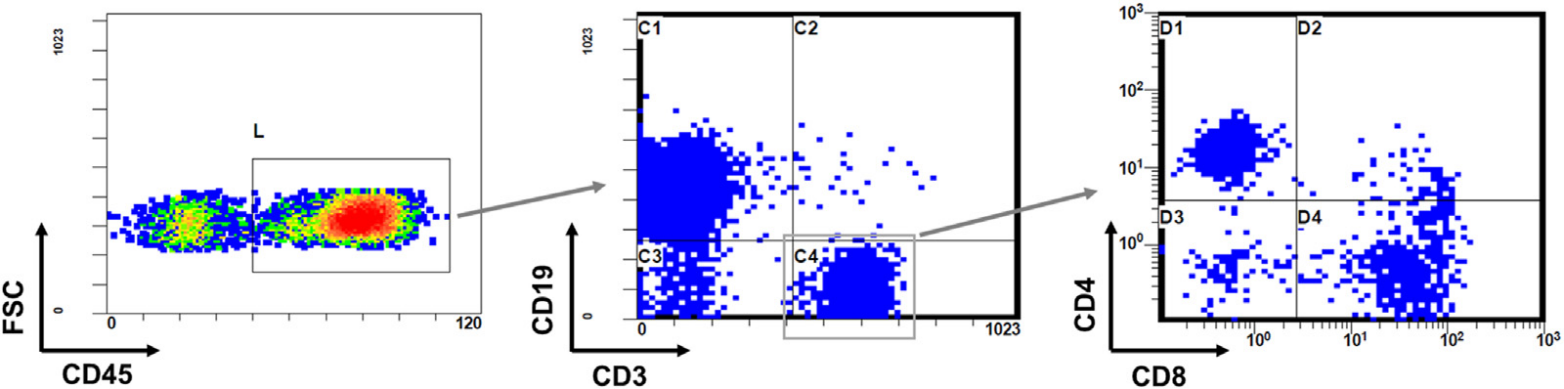
Engraftment of human cells in the peripheral blood of humanized NOG-Iab KO, HLA-DR 0405 Tg mice. Peripheral blood cells from humanized NOG-Iab KO, HLA-DR 0405 Tg mice 26 weeks after transplantation were stained with anti-human CD45, anti-human CD3, anti-human CD4, anti-human CD8, and anti-human CD19 antibodies. In the peripheral blood mononuclear cell fraction, populations of human T- and B-cells were identified among human CD45+ cells, and populations of human CD4+ or CD8+ T-cells were identified among human CD4+ cells.

After transplanting CD34-positive HSCs into 14 NOG-Iab KO, HLA-DR 0405 Tg mice (8 males and 6 females), engraftment was analyzed by multicolor flow cytometry. In all mouse peripheral blood samples, a population of human B-cells appeared two months after transplantation, and human T-cells appeared four months after transplantation. None of the 14 hu-HSC NOG-Iab KO, HLA-DR 0405 Tg mice developed symptoms of GVHD, such as bodyweight loss, hair-loss or hunched gesture, during the 26 weeks after transplantation. Mice were euthanized 26 weeks after transplantation. At the time of dissection, human T- and B-cell populations were found in all mouse peripheral blood samples, and both CD4 + and CD8 + T-cell populations were identified in the human T-cell population (Figure 1).

**Figure 2:**
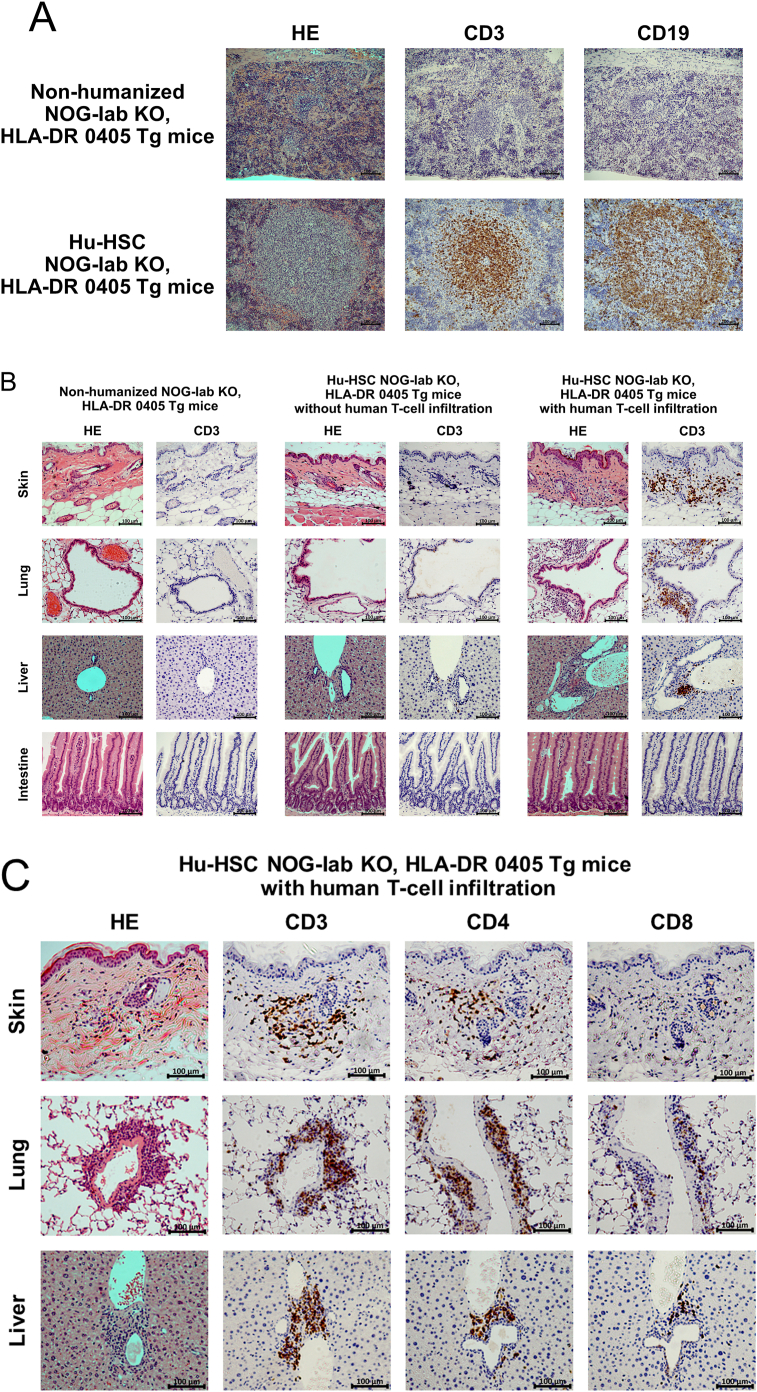
Analysis of histological findings in humanized NOG-Iab KO, HLA-DR 0405 Tg mice. Hematoxylin-eosin and immunohistochemical staining of (A) spleen, (B) (C) skin, lung, and liver in humanized (hu-HSC) and non-humanized NOG-Iab KO, HLA-DR 0405 Tg mice. Anti-human CD3, human CD19, human CD4, and human CD8 antibodies were used for immunohistochemical staining. The black line represents 100 μm. (A) Follicular-like structures were found in the spleen of hu-HSC NOG-Iab KO, HLA-DR 0405 Tg mice, which were occupied by both human T- and B-cells. (B) Histological findings of human T-cell infiltration were found in the dermis of the skin, around the bronchioles and blood vessels of the lungs, and around the portal vein of the liver, however, they were not found in the gastrointestinal tract. (C) Infiltrated human T-cells were predominantly CD4+.

**Figure 3:**
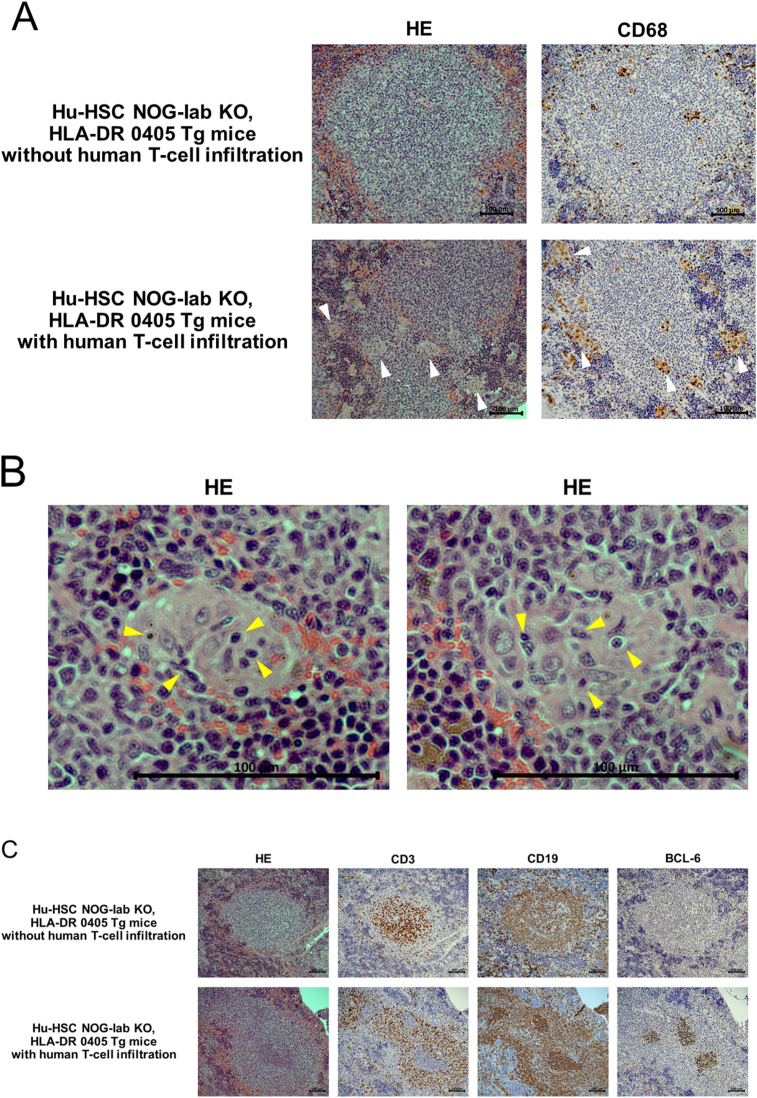
Histological findings of B-cell activation in the spleen of humanized NOG-Iab KO, HLA-DR 0405 Tg mice. Hematoxylin-eosin and immunohistochemical staining of the spleen in humanized (hu-HSC) NOG-Iab KO, HLA-DR 0405 Tg mice are shown. Anti-human CD68, human CD3, human CD19, and human BCL-6 antibodies were used for the immunohistochemistry. The black line represents 100 μm. (A) In hu-HSC NOG-Iab KO, HLA-DR 0405 Tg mice with human T-cell infiltration, tingible body macrophages (TBM) were increased in the spleen (white arrow). (B) Cytophagocytosis was observed in the cytoplasm of the TBM (yellow arrow). (C) Clusters of BCL-6 positive B-cells were found in the follicular-like structure of the hu-HSC NOG-Iab KO, HLA-DR 0405 Tg mice with human T-cell infiltration.

**Figure 4:**
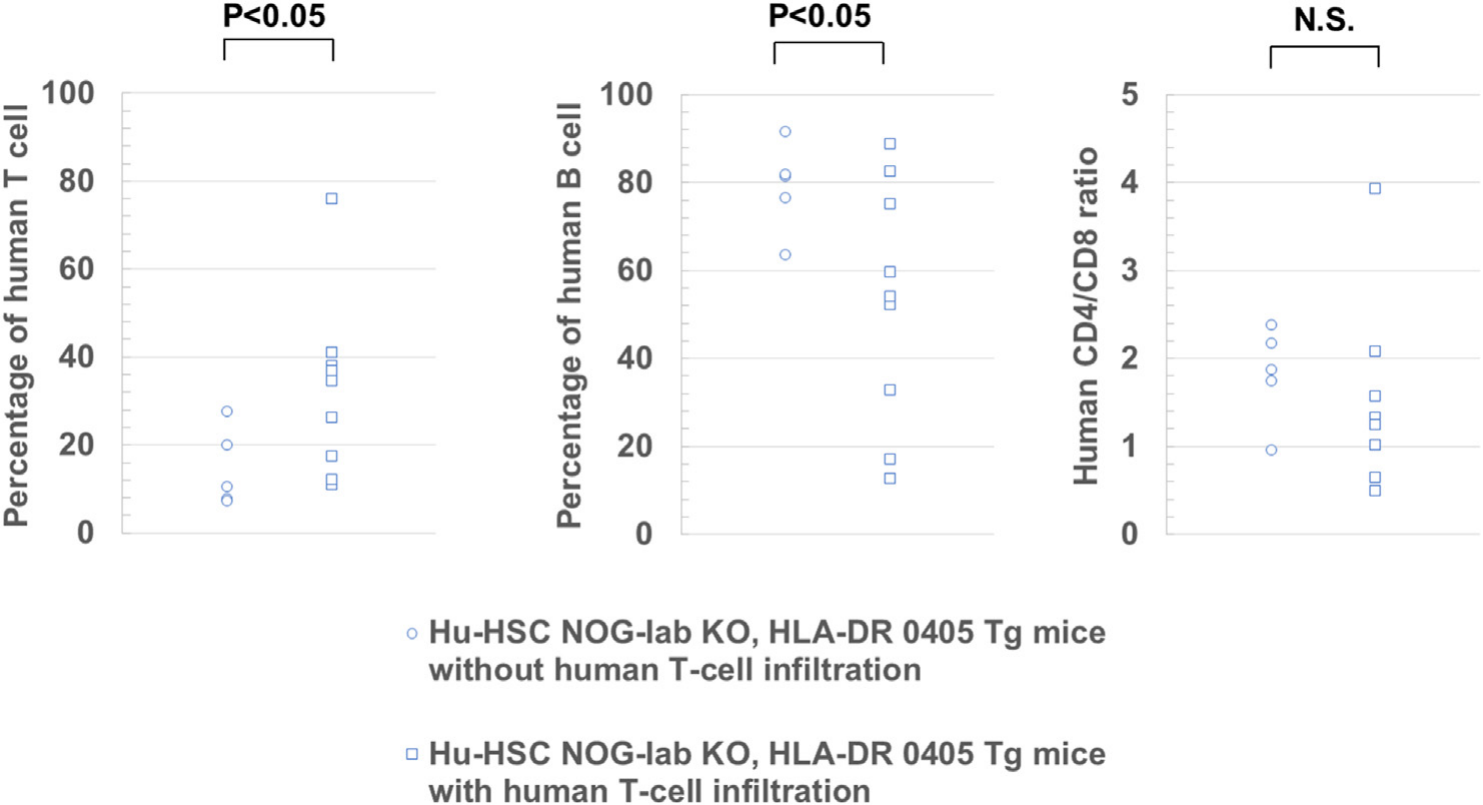
Analysis of human T- and B-cell ratios and CD4/CD8 ratios in humanized NOG-Iab KO, HLA-DR 0405Tg mice. Percentage of human T-cells and human B-cells in peripheral blood human mononuclear cells of humanized NOG-Iab KO, HLA-DR 0405Tg mice and the CD4/CD8 ratio. Peripheral blood cells from humanized NOG-IabKO, HLA-DR 0405 Tg mice at dissection were stained with anti-human CD45, anti-human CD3, anti-human CD4, anti-human CD8, and anti-human CD19 antibodies. Welch's t-test was used to detect statistically significant differences.

We performed histological analysis on the spleen, skin, lungs, liver, and gastrointestinal tract of hu-HSC NOG-Iab KO, HLA-DR 0405 Tg mice and compared these to samples from negative controls. In the spleen, lymphoid tissue around the blood vessels developed and imitated a follicular-like structure in all 14 mice experimental samples. Human T- and B-cells were mixed in the follicular-like structure (Figure 2A); this was markedly different to the negative control. In 9 of the 14 hu-HSC NOG-Iab KO, HLA-DR 0405 Tg mice (4 males and 5 females), histological features, human T-cell infiltration, were found in the skin, lungs, and liver; however, none were observed in the gastrointestinal tract (Figure 2B). The skin showed epidermal thickening and intradermal infiltration of human T-cells; however, there was no indication of increased dermal collagen fibers. The lungs showed peribronchiolar and perivascular infiltration of human T-cells, predominantly CD4 + cells; however, no bronchiole fibrotic stenosis was observed. The liver showed periportal infiltration of human T-cells; however, no periportal fibrosis or fibrotic stenosis of the bile duct was shown. In these organs, CD4+ cells were the predominant type among the infiltrating human T-cells (Figure 2C). Nine mice spleens, which had shown human T-cell infiltration, all had tingible body macrophages (TBM) found in them; however, the spleen of the remaining five mice did not have any TBM (Figures 3A, 3B). In four of the nine mice with human T-cell infiltrated spleens, clusters of BCL-6 positive B-cells were found in the follicular-like structure (Figure 3C). The percentage of human T-cells and B-cells in peripheral blood human mononuclear cells at the time of dissection was analyzed. The percentage of human T-cells was higher in mice with the infiltration of T-cells than in mice without infiltration, and the percentage of human B-cells was relatively lower. There was no difference in the CD4 versus CD8 ratio between the groups (Figure 4).

### 3.2 Analysis of serological findings

**Figure 5:**
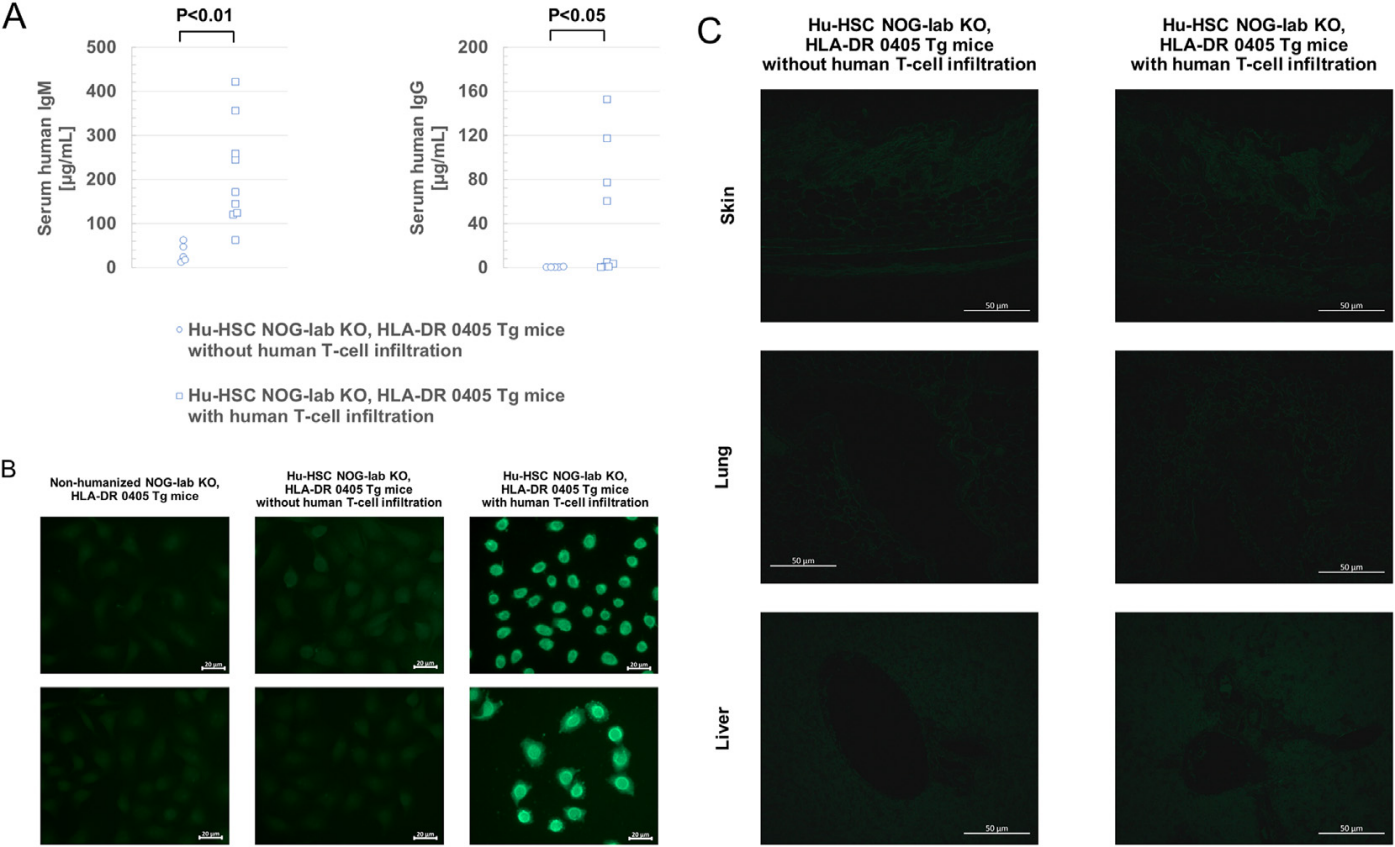
Analysis of serum human immunoglobulin levels and antinuclear antibodies in humanized NOG-Iab KO, HLA-DR 0405 Tg mice. (A) Human IgM and IgG levels in serum after the dissection of humanized (hu-HSC) NOG-Iab KO, HLA-DR 0405 Tg mice. Welch's t-test was used to detect statistically significant differences. (B) Detection of human IgG antinuclear antibodies by indirect immunofluorescence antibody method. In hu-HSC NOG-Iab KO, HLA-DR 0405 Tg mice with human T-cell infiltration (n = 7), 320-fold diluted sera were used (right panel). In non-humanized NOG-Iab KO, HLA-DR 0405 Tg mice (n = 3) and hu-HSC NOG-Iab KO, HLA-DR 0405 Tg mice without human T-cell infiltration (n = 5), 80-fold diluted sera were used (left and central panels). The white line indicates 20 μm. (C) Antibody deposition in the skin, lung, and liver of hu-HSC NOG-Iab KO, HLA-DR 0405 Tg mice was determined by immunofluorescence staining with Alexa Fluor® 488-conjugated anti-human IgG. The white line indicates 50 μm.

To evaluate the humoral immune system of hu-HSC NOG-Iab KO, HLA-DR 0405 Tg mice, we examined human immunoglobulin levels and detected for the presence of ANA in their sera during dissection. Both serum human IgM and human IgG levels were markedly higher in mice with human T-cell infiltration than in mice without infiltration (Figure 5A). Almost no human IgG was detected in the sera of mice without human T-cell infiltration. Human IgG ANA were detected in the sera of seven of the nine mice (two males and five females) with human T-cell infiltration; however, ANA were not detected in the sera of five mice that did not have infiltrating human T-cells (Figure 5B). All detected ANA showed a homogeneous or peripheral staining pattern and a high titer (320 times). To analyze whether autoantibodies were directly involved in organ lesions, we examined antibody deposition in the skin, lungs, and liver of humanized mice. We did not observe antibody deposition in any of the organs of mice with and without human T-cell infiltration (Figure 5C).

## 4 Discussion

Humanized mice are produced by transplanting and engrafting human HSCs into immunodeficient mice; this enables the human immune system to be reconstituted in mice. In particular, hu-HSC NOG mice have been used previously to study viruses that infect human lymphocytes, such as human immunodeficiency virus (HIV) and Epstein-Barr virus (EBV) [15, 16]. Unfortunately, there have been instances when humans have experienced adverse effects with medication previously found to be safe in animal preclinical studies [17]. Therefore, humanized mice are expected to help solve this problem.

There are two types of GVHD, namely, acute and chronic. Acute GVHD develops when donor-derived T-cells recognize and attack recipient cells as non-self [18]. On the contrary, the pathogenic mechanism of chronic GVHD is not completely understood. In the clinical practice of chronic GVHD, cases of transition from acute GVHD and cases of de novo onset have been reported. Risk factors for onset are not completely consistent between acute and chronic GVHD, and chronic GVHD is not simply the chronicity of acute GVHD [19]. Existing experimental mouse models of chronic GVHD, such as parent to F1, B10.D2→BALB/c, LP/J→C57BL/6, DBA/2→BALB/c, and C57BL/6→BALB/c, have limitations [20, 21, 22, 23]. In these cases, a sufficient amount of peripheral blood T-cells (including splenocytes) were administered to mice, and GVHD developed 1–2 months after administration. We consider that acute GVHD developed and transitioned to chronic GVHD. In humanized mice [8, 9, 10, 11, 12, 13], purified human CD34+ cells, with almost no CD3+ cells, are transplanted into immunodeficient mice to create them, in order to prevent the development of acute GVHD. In the previously reported chronic GVHD in humanized mice [8, 9], GVHD develops 4–5 months after transplantation of high-purity CD34-positive cells containing almost no peripheral blood T-cells, suggesting that GVHD developed by a mechanism different from that in the existing experimental mouse models. Therefore, humanized mice are worth applying in the study of chronic GVHD. However, in previous reports on chronic GVHD in humanized mice, the clinical features of chronic GVHD could not be fully reproduced, because B-cell activation and ANA production did not occur. To ameliorate the above problems and establish a new model of chronic GVHD in humanized mice, we investigated whether NOG-Iab KO, HLA-DR 0405 Tg mice develop chronic GVHD after HSC transplantation. In a previous study [14], transplantation of HLA-DRB1∗0405-negative UCB-derived CD34+ cells into NOG-Iab KO, HLA-DR 0405 Tg mice resulted in poor reconstitution of the humoral immune system; therefore, HLA-DRB1∗0405-positive UCB was used in the present study. In this study, the time of appearance of human lymphocytes in the peripheral blood of humanized mice was consistent with previously reported lymphocyte differentiation in humanized mice [13, 24]. We considered that human CD34-positive cells were well engrafted, and B- and T-cells differentiated from them.

In the previously reported chronic GVHD in humanized mice [8, 9], GVHD symptoms, such as gingivitis and hair loss, appeared 16–24 weeks after transplantation; therefore, our observation period was set to 26 weeks after transplantation. In this study, no GVHD symptoms were identified in humanized mice 26 weeks after transplantation; however, histological abnormalities were found in the skin, lungs, and liver, similar to those found in a previous study. Fibrotic lesions, which are usually characteristic of chronic GVHD, were not observed in this study, since the observation period was short. In addition, although mice were irradiated before transplantation in previous reports, we could not perform irradiation before transplantation due to equipment problems. It has been pointed out that irradiation before transplantation causes systemic cytotoxicity, activates the innate immune system, and is involved in the development of chronic GVHD [25]. In this study, the lack of irradiation may have affected the onset of chronic GVHD. Moreover, neither this study nor any previous reports showed abnormalities in the pathological findings of the gastrointestinal tract, which may be related to the reconstruction of the intestinal immune system. Nochi [26] reported that gut-associated lymphoid tissue (GALT) was not formed in the hu-HSC NSG mice; thus, the reconstruction of the intestinal immune system was insufficient. The formation of GALT requires cell populations (cryptopatches) present in the intestinal crypt, with IL-7 also essential for their maintenance [27]. Since NSG mice and NOG-Iab KO, HLA-DR 0405 Tg mice were common γ-chain-deficient mice, we determined that IL-7 receptor dysfunction caused hypoplasia of the cryptopatch and GALT. Thus, it was speculated that the intestinal immune system was not sufficiently reconstructed in humanized mice using either NSG or NOG-Iab KO, HLA-DR 0405 Tg mice; moreover, no pathological findings were observed in the gastrointestinal tract of these mice. According to a previous study in which NOG or NSG mice were administered human peripheral blood or cord blood mononuclear cells, human T-cells transplanted caused organ lesions approximately one month after administration, and the gastrointestinal tract, skin, liver, and lungs were the target organs [9, 14]. It is interesting that there was a difference in the presence or absence of gastrointestinal lesions between CD34-positive cell transplantation and peripheral blood mononuclear cell transplantation. This is another evidence that the pathogenesis of organ lesions after CD3 cell transplantation is different from that after CD34 positive cell transplantation. In chronic GVHD that develops after CD 34 positive cell transplantation, organ lesions develop after a certain period, about half a year, rather than a short period of one month after transplantation. This suggests that the organ lesions in this study were not derived from contaminated human T-cells in the transplanted cells. It should be considered that the organ lesions in this study were early changes in chronic GVHD that develops after CD 34 positive cell transplantation.

In the spleen of nine hu-HSC NOG-Iab KO, HLA-DR 0405 Tg mice with human T-cell infiltration, histological findings of TBM were common; moreover, clusters of BCL-6-positive B-cells were formed some of these. Clusters of BCL-6-positive B-cells indicated germinal center formation in previous studies [28, 29]. TBM emerges during germinal center formation [30, 31] and has a physiological role in phagocytosing apoptotic low-antigen-affinity B-cell clones [32]. From our results, it was suggested that the histological findings of the spleen indicated the activation of B-cells. Furthermore, serum human IgM and IgG levels were markedly higher in hu-HSC NOG-Iab KO, HLA-DR 0405 Tg mice with human T-cell infiltration than in those without human T-cell infiltration; moreover, histological findings detected human T-cell infiltration and good immune reconstitution of donor B-cells. This suggests a relationship between immune rearrangement of donor B-cells and the development of chronic GVHD. In the mouse model (allogenic transplantation between mice), it has been reported that germinal center formation and IgG production via donor B-cells were required for the development of chronic GVHD [33]. On the contrary, there was a report that the development of chronic GVHD was associated with the destruction of lymphoid follicles and germinal centers, and IgG produced by transplanted donor B-cells was involved in the destruction of lymphoid follicles and germinal center structures [34]. There were differences in the relationship between the onset of chronic GVHD and germinal center formation in each mouse model. It is necessary to clarify the changes in germinal center structure during the progression of organ lesions in hu-HSC NOG-Iab KO, HLA-DR 0405 Tg mice.

Furthermore, we revealed that high-titers of ANA appeared in the sera of hu-HSC NOG-Iab KO, HLA-DR 0405 Tg mice that also had human T-cell infiltration. However, no antibody deposition was observed in any of the tissues with human T-cell infiltration. This suggests that ANA was not directly involved in organ lesions and that the origin of organ lesions in this study was not inflammation due to immune complex deposition. This study is the first report on B-cell activation and autoantibody production in humanized mice with chronic GVHD. The presence of ANA indicates the activation of autoreactive B-cells. Autoreactive B-cells are closely involved in the pathophysiology of chronic GVHD [35, 36, 37]. This study differs from the previously reported chronic GVHD in humanized NSG mice in that of the activation of autoreactive B-cells; further research should clarify how it affects organ lesions in the long term.

Some limitations exist in this study. The first is that the observation period after transplantation was short. We only evaluated the early stages of chronic GVHD, and it is not yet clear whether fibrotic lesions, which are characteristic of chronic GVHD, occur in each organ. Long-term observation after transplantation is required. The second limitation is that the mice were not irradiated before transplantation. It is necessary to investigate the importance of irradiation before transplantation and onset of chronic GVHD. If irradiation causes earlier onset or increases the incidence of chronic GVHD, it will improve the establishment of a new mouse model. Third, we focused on organ lesions and ANA, and did not perform a functional assessment of human lymphocytes. An analysis of the activation of a subset of human T- and B-cells in peripheral blood and spleen is important in investigating the pathophysiology of chronic GVHD.

In conclusion, the present study demonstrated that NOG-Iab KO, HLA-DR 0405 Tg mice can be used as a new model of chronic GVHD in humanized mice. The chronic GVHD that developed in hu-HSC NOG-Iab KO, HLA-DR 0405 Tg mice was different from conventional models in that B-cell activation and ANA production simultaneously occurred. Establishing a model of chronic GVHD using hu-HSC NOG-Iab KO, HLA-DR 0405 Tg mice can be used to elucidate the pathophysiology of this disease and any potential therapeutics in the future.

## Declarations

### Author contribution statement

Hiroshi Tsuzuki: Conceived and designed the experiments; Performed the experiments; Analyzed and interpreted the data; Wrote the paper.

Yasuko Nagatsuka: Performed the experiments; Analyzed and interpreted the data.

Mitsuhiro Iwata, Noboru Kitamura, Yosuke Nagasawa, Hideki Nakamura: Analyzed and interpreted the data.

Taro Matsumoto, Ryoji Ito, Takeshi Takahashi, Mamoru Ito: Contributed reagents, materials, analysis tools or data.

Masami Takei: Conceived and designed the experiments; Analyzed and interpreted the data.

### Funding statement

This work was supported by 10.13039/501100001700Ministry of Education, Culture, Sports, Science and Technology (MEXT)-Supported Program for the Strategic Research Foundation at Private Universities, 2015–2019.

### Data availability statement

Data included in article/supplementary material/referenced in article.

### Declaration of interests statement

The authors declare no conflict of interest.

### Additional information

No additional information is available for this paper.
